# Some Omissions in the National Insurance Bill

**Published:** 1911-06-10

**Authors:** 


### HOSPITALS AND STATE INSURANCE.

### SOME OMISSIONS IN THE NATIONAL INSURANCE BILL.

FROM A CORRESPONDENT.

It is not to be expected that a Bill which introduces an entirely new idea into legislation should be perfect in all its details, and thus it happens that, while the National Insurance Bill has had, on the whole, an exceptionally warm welcome, there have come, from many sides, criticisms of things which, in the opinion of the critics, have been done amiss, and other things which have been omitted. And it is good that such criticisms should be uttered, and that the Government should be assured that they are uttered in a friendly spirit. It may be that the complainers have misunderstood the meaning of some of the provisions of the Act, but in an Act like this it is important that every point should be as clear as possible, or it may chance that in its future interpretation at the hands of judges injustice may be done where nothing but absolute fairness was meant. Thus, there is arising a feeling among midwives that the maternity provisions of the Act will favour medical men at their expense. This assuredly was never meant by the framers of the Act, nor do we think that in an age when feminist influence is so powerful and feminine voices are so persistent and so loud, there is much danger of the woman worker having her interests neglected. At the same time it should be made clear that the trained woman who, as nurse or midwife, gives her care to mother and infant, should have her status and claims upheld by the State, even as the man who does the same. The status is not the same and the claims are not the same; but status and claims there are which cannot be ignored.

On other points questions are arising on the part of women. In the first place, women are complaining of the deductions to be made from their wages. Unreasonably perhaps, since in sickness they are to receive substantial benefit, but quite comprehensibly, when one reflects that the average wage earned by women in this country is 7s. 6d. a week. As a fair number of workers earn more than this, it is obvious—as, indeed, we know—that many must earn less, and to deduct threepence a week towards State insurance may quite comprehensibly seem to the less fortunate that the State, in order to help them when they are ill, is trying to starve them when they are well. There is a point to which the living wage—and still more the wage which does not sufficiently provide for living—cannot be reduced, and it may evolve in the end that employers will have to pay indirectly the employees' contribution to the State fund as wel as their own. But meanwhile, until conditions settle themselves, it is obvious that a contribution from women who at their present rate of wages can hardly keep themselves, must cause great hardship.

Again, there is the question of giving contribution from which no benefit to the insured accrues. The objection of the uneducated to this is very clear. With people who have a certain amount of intelligence it is obvious that if John Smith, who falls ill within a year of his insurance is to be maintained, it must be partly through the contributions of Thomas Brown and Henry Robinson, who have been paying for years and have not required to claim anything. This underlying principle of insurance is not to be adhered to too strictly in the State scheme, which draws its funds from no other than beneficiaries, but to the poorest of workers even the modest tax which Government demands may seem too much—especially as these are among the number who at present, when ill, fall back upon the parish doctor and outdoor relief, or, in serious cases, go to the Poor-Law Infirmary. To such as these the contribution to the national fund is an immediate loss without even a possible gain. In country districts there may linger some dislike, born of hereditary pride and independence, of parish relief, but in the metropolis at least the "guardians" are regarded as friends and "the infirmary" as a home of refuge.

And on the other side will be found some who resent the employer's contribution—and perhaps take it out of the wages of the employed. This is a factor which must be reckoned with in the case of either sex, and always it will be the worst paid—those who can the least afford to lose a penny—who will be compelled to submit to such exaction, for it is they who can least afford to fight and stand out for better terms. While this is true of both sexes, it has a special importance in the case of women whose earnings as a rule are small, and who have an ingrained fear of "quarrelling with their bread and butter." It is doubtful if any legislation can touch a moral problem like this.

There is another point on which women may legitimately feel themselves aggrieved. It is the natural destiny of women to marry, and the bulk of them find it sooner or later—in the working classes as a rule pretty early. But for years before their marriage the girls of that class will have been engaged on wage-earning work, and paying contributions to the national insurance fund from which in many cases they will have received no benefit. And if, on marriage, they retire—as one would always wish them to do—from work outside the home, their premiums are all lost; they have no longer any claim. It is to be expected that girls will resent being mulcted in their small wages during their most vigorous years without any prospect of profiting by their contributions at a later date. Whether or not it would be possible to permit a wife to continue her contributions after marriage if her husband would give the employer's contribution, and thus qualify for sick benefit, is a question which might be debated. But it would remove a grievance which will be much felt and much talked about. If no arrangement is made whereby a woman's contributions are to profit her after her marriage she will be tempted to continue with her outside work, with all the usually unfortunate results of this on home, husband, and offspring. There is also a general feeling that the maternity benefit should, after due payment has been made to the doctor or nurse in attendance, be given in cash to the wife. It is true that there are cases in which it might be more beneficial that the balance of the thirty shillings maternity benefit, left after paying for medical attendance, should be expended by outside persons, but surely such cases are not the rule. To enforce such a law is to imply that all husbands in the poorer classes are drunkards or selfish brutes, who would grudge the wife and baby the care and nourishment they need. This assumption that the poor do not know their own needs is one of the frequent follies of voluntary charity—food is given where coals are wanted, or where some help with the rent would lift a load of anxiety from the sufferers' mind. We should expect more common-sense from State officials, but in the majority of cases we might safely trust the poor to judge of their own greatest needs. We quite realise that the doctor or midwife should be paid before the balance is handed over, as otherwise the money might dribble away before the accoucheur's dues were paid, but as to the rest of the money, which is to go in comforts for mother and child, the mother may be regarded as the best judge of what is required.

Mr. Lloyd George excuses the omission of married women who are not wage-earners from the benefits of the Act by dwelling on the difficulty of preventing malingering. But surely this can be dealt with by the simple expedient of paying no sick benefit unless a doctor's certificate is produced. We can hardly imagine that our medical men would give certificates under false pretences.

A difficult point arises—though not with regard to sickness benefit—with regard to widows. If they return to the wage market they can again insure—that is only fair. But the widow is a person whom, if she has young children, one does not want to see going back to factory or workshop. And, indeed, she often tries to avoid this by becoming that worst-paid of all creatures, the so-called "home-worker." After all she can, in the midst of her box-making or fur-pulling, give an eye to the children, and that makes up in her eyes for the meagreness of her wages. The present Poor Law method—which is the only one that has tried to deal with this problem—is to take the elder children into the Poor Law schools and leave the widow to work for herself and the youngest—the one who most needs that she should be at home. The results are not altogether happy for the mother nor good for the youngest child, while the cost of the elder ones in the schools is a thing which would irritate even the long-suffering ratepayer if he knew of it. One would fain see a national scheme which would make some provision for fatherless children of school age, enabling them to remain at home, brought up with the parent whom they still possess, and given a weekly income which should be sufficient to keep them above destitution. Our present methods, which rob the poor widow of her children when fate takes her husband from her, may be regarded as an accidental form of suttee, but not much of an improvement on the Oriental one. Cannot widowhood be provided for in our national insurance?

### THE NATIONAL INSURANCE BILL.

